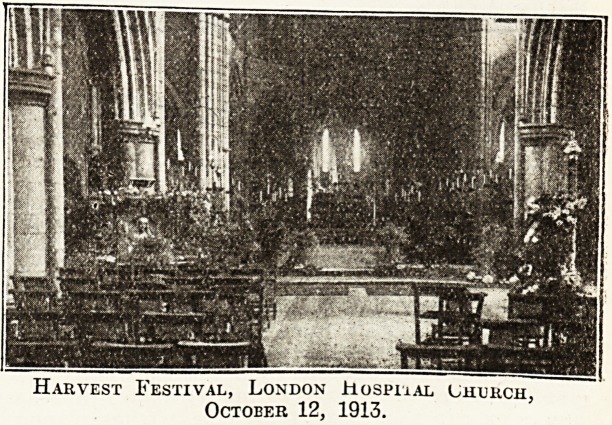# The Central Point of the Chaplain's Work

**Published:** 1914-04-18

**Authors:** B. Rees


### SOME CHAPELS OF FAMOUS HOSPITALS.

### The Central Point of the Chaplain's Work.

By the Rev. B. REES.

The relation existing between the Church and her ministers with the tending of the sick poor has always been a close one. The bishops of the early Christian Church, acting apparently upon the Apostolic injunction to heal the sick, erected "Houses of Charity," afterwards known as hospitals, which were under the management of the priests and deacons. These institutions were soon suppressed, and with the advance of learning and science the priest-physician ceased to exist. As late as 1500, however—the barber-surgeon period—we read of permission to practise medicine being in the hands of the bishops, while the healing of the sick was left to those specially trained for the work. The clergy meanwhile devoted themselves entirely to spiritual ministrations as hospitallers—a name derived from the Order of religious knights of Jerusalem—or chaplains. It is, therefore, of more than passing interest to glance at what

A Monk Physician of the Fifteenth Century.

is largely the centre of the work of the chaplains, or, in other words, at the chapel of the modern hospital.

St. Bartholomew's Hospital.

The hospital church, dedicated to St. Bartholomew-the-Less, standing within the precincts of the hospital, is the survivor of three ancient chapels. After escaping the Great Fire of London it was rebuilt and restored both in 1789 and 1823. The roof presents an example of the early experiments of the Gothic revival, and within its walls numerous monuments are to be found, among them a brass of Robert Balthrope, Sergeant of the Surgeons to Queen Elizabeth, and a memorial to Sir James Paget. In the reign of Henry VIII. the Vicar of St. Bartholomew-the-Less was made *ex-officio* the hospitaller; the office is now held by the Rev. H. S. Close. There is a legend that on certain occasions shadowy figures in antique garb are to be seen filling the pews, which may probably be traced to the founding of the hospital by a Canon Regular of St. Austin and his monks.

An interesting example of the close relationship of Church and medicine is to be seen in the Charge given to William Harvey when appointed as physician to St. Bartholomew's, in which he is required to cause "the Hospitaller, Matron, or Porter" to bring to him those in need of treatment. This shows that the hospitaller of

Church of St. Bartholomew-the-Less.

his time had a share in the bodily as well as in the spiritual needs of the patients.

Westminster Hospital.

Though the third oldest hospital in London (1719), the present Westminster Hospital is the fifth building; in

Ciborium made circa 1630-40. Now Used in Guy's Hospital Chapel.

consequence there is little ecclesiastical or other history surrounding its chapel, which was built in 1886. It is of a worthy design, and bears upon its walls numerous brasses and memorials which tell the story of lives willingly laid down in the call of duty. The beauty of the interior is much enhanced by the possession of five of the finest of Kemp's stained-glass windows; there is also an excellent little two-manual organ, which was erected with the help and advice of Sir Frederick Bridge. A custom which perhaps is worthy of note is that the chapel choir go the rounds of the several wards of the hospital in procession every Sunday evening at the time of service.

### Guy's Hospital.

Built in 1774, the chapel of Guy's Hospital is a fine example of the style of the period, the roof being especially striking. In 1725 the first chaplain was appointed at the salary of £80 (equalling about £400 a year at the present day), and the most famous hospitaller was F. D. Maurice, who held the post in 1834. The year 1858 saw the chapel rearranged as it is to-day, and as a memorial to Mr. Hunt, the second great benefactor of the hospital, five stained-glass windows were placed in the east end.

The object of central interest within the chapel is the memorial to the hospital's founder—Thomas Guy—in which he is depicted in his livery gown holding out his

Guy's Hospital Chapel.

left hand to help a poor invalid who is upon the ground, while with his right he points to the hospital behind him and to a sufferer who is being carried on a litter to the wards.

Upon the pedestal of the monument appears the following :—

UNDERNEATH ARE DEPOSITED THE REMAINS OF
THOMAS GUY,
CITIZEN OF LONDON, MEMBER OF PARLIAMENT, AND THE SOLE
FOUNDER OF THIS HOSPITAL IN HIS LIFE-TIME.
IT IS PECULIAR TO THIS BENEFICENT MAN TO HAVE
PERSEVERED DURING A LONG COURSE OF PROSPERITY AND
INDUSTRY, IN POURING FORTH TO THE WANTS OF OTHERS, ALL
THAT HE HAD EARNED BY LABOUR OR WITHHELD FROM
SELF-INDULGENCE.
WARM WITH PHILANTHROPY AND EXALTED BY CHARITY HIS
MIND EXPANDED TO THOSE NOBLE AFFECTIONS WHICH GROW
BUT TOO RARELY FROM THE MOST ELEVATED PURSUITS.
AFTER ADMINISTERING WITH EXTENSIVE BOUNTY TO THE
CLAIMS OF CONSANGUINITY
HE ESTABLISHED THIS ASYLUM FOR THAT STAGE OF LANGUOR
AND DISEASE TO WHICH THE CHARITY OF OTHERS HAD NOT
REACHED; HE PROVIDED A RETREAT FOR HOPELESS
INSANITY AND RIVALLED THE ENDOWMENTS
OF KINGS.
HE DIED THE 27TH OF DECEMBER, 1724, IN THE 80TH YEAR
OF HIS AGE.

### Middlesex Hospital.

Although the Middlesex Hospital was founded in 1745, and the oldest part of the present building built in 1755, it was not until 1890—and then as a memorial to the late Major Ross, chairman of the weekly board of governors for twenty years, and to others who had served the hospital—that the chapel was begun. In the year following the chapel was opened, but much of the originally proposed interior decoration remained to be done. This, however, is now completed. In the present writer's opinion it is one of the most beautiful of chapels. The choir and sanctuary are vaulted, and the vaulting is overlaid with gold mosaic. Cipollino marble covers the lower part of the walls, and above the dado thus formed are alabaster and bands of mosaic at intervals. The altar is of marble with jasper and onyx panels and mosaic

Middlesex Hospital Chapel.

work; the credence table is formed by an arched recess of alabaster and mosaic, and the piscina is of similar materials. Two pilasters of Brêche sanguine marble with carved alabaster capitals support the chancel arch, and the decoration of the nave is, on the whole, a continuation of the walls of the sanctuary. In the east wall over the chancel arch are the symbols A and Ω and on the north above the windows appear two medallions; on the south three medallions with figures of saints and Evangelists. The west wall has a memorial tablet to Major Ross; opening also from it is the organ chamber, having a fine parapet of alabaster with panels of jasper, onyx, and agate. In the ante-chapel are to be seen a series of oblong white marble frames with inscriptions to commemorate those who during their lifetime have been connected with the hospital. The reredos is a triptych in wood, richly decorated; it represents the Risen Lord Adored by Saints and Angels. The lectern is an eagle in white alabaster attached to a low wall which encloses the choir.

### The Old and New King's College Hospital.

The old King's had a very close connection with church life, for a portion of it was built upon what was once a part of the old churchyard of St. Clement Danes, and built into the old hospital walls was to be found the memorial to the humorist "Honest Joe Miller," recently alluded to in The Hospital, upon whose stone appears the following :—

IF HUMAN WIT AND HONESTY COULD SAVE
THE HUMOROUS WITTY HONEST FROM THE GRAVE,
THE GRAVE HAD NOT SO SOON A TENANT FOUND
WHOM HONESTY AND WIT AND HUMOUR CROWNED.
COULD BUT ESTEEM AND LOVE PRESERVE OUR BREATH,
AND GUARD US LONGER FROM THE STROKE OF DEATH,
THE STROKE OF DEATH ON HIM HAD LATER FELL
WHOM ALL MANKIND ESTEEMED AND LOVED SO WELL.

The new King's Chapel, dedicated on October 2 of last year by the Chaplain, the Bishop of Southwark, is a worthy and dignified building with the keynote of

The Chapel, King's College Hospital, Denmark Hill, S.E.

simplicity in its decorative scheme. A suitable use of marbles has provided a beautiful chancel, and the altar, which possesses a rich dossal, can be seen from every part of the building. The organ has been removed from the old site in Portugal Street, and now finds after enlargement a place at Denmark Hill, as do also the stained-glass windows and pulpit as well as the valuable painting by Juan Juanes. The ornaments and fittings of the chapel are in keeping with the rest of the building, and are largely the result of the generous effort of the sister-matron and the nursing staff. The chapel has accommodation for 250 persons.

### Great Ormond Street Hospital.

The Hospital for Sick Children boasts a most beautiful chapel which was erected by Mr. William Barry in memory of his wife, who died in 1872. The pillars of this gem among places of worship are of polished marble with gilded capitals and bases of alabaster, with which material the walls also are covered. Richly ornamented brass gates admit to the sacrarium, and the chapel is lighted with brass candelabra (now fitted with electric light). The floor is of mosaic, and the seats of ebonised wood, while on either side of the chapel are frescoes representing "Christ Blessing Little Children" and "The Good Shepherd." The windows are of richly stained glass, and present pictures of Our Lord's Infancy and Childhood, and of the child-saints, Timothy, Samuel, and John the Baptist. The roof also is handsomely painted and gilded, and each Sunday the chapel is decorated with beautiful flowers. The acting chaplain is the Rector of St. George the Martyr, Queen Square, W.C., in which parish the hospital is situated.

### Victoria Park Hospital.

In 1857 an offer was made to the council of the hospital by Mr. John Derby Allcroft to build a chapel. This offer was readily accepted, and the foundation stone was laid in the following year. In April 1860 the building was completed and opened; it is somewhat large and thus lacks the compactness of the average hospital chapel—in fact, it is stated that if all connected with the hospital—patients and staff—were able to attend, there would be still room for a considerable number more. It is in the decorative style, and its architecture is pleasing. Historically the chapel is of interest as having been built upon a piece of the garden of the palace of Bishop Bonner, and tradition points out a certain mulberry tree standing near as the tree under which Bonner used to sit in the cool of the summer evenings to plan out fresh schemes for the holocausts of his enemies.

### London Hospital : Secretary and Chaplain Combined.

The hospital chapel proper is now merely one bay and the chancel of the original chapel. It possesses a fine stained-glass window in the west end, but apart

Harvest Festival, London Hospital Church, October 12, 1913.

from that there is little of interest connected with it. The reduction of the original chapel was occasioned by the need of room for new departments in the hospital and the building of the hospital church of St. Philip, Stepney, a magnificent building in the Continental style. There has been a chaplain since 1740, and curiously enough at one time the office of secretary and chaplain was a combined one, and part of the duty of the holder was to bury any patient who died in the hospital in a field near the institution, which now forms the hospital garden and tennis courts. At such burials all patients who could get up were expected to attend!

## Figures and Tables

**Figure f1:**
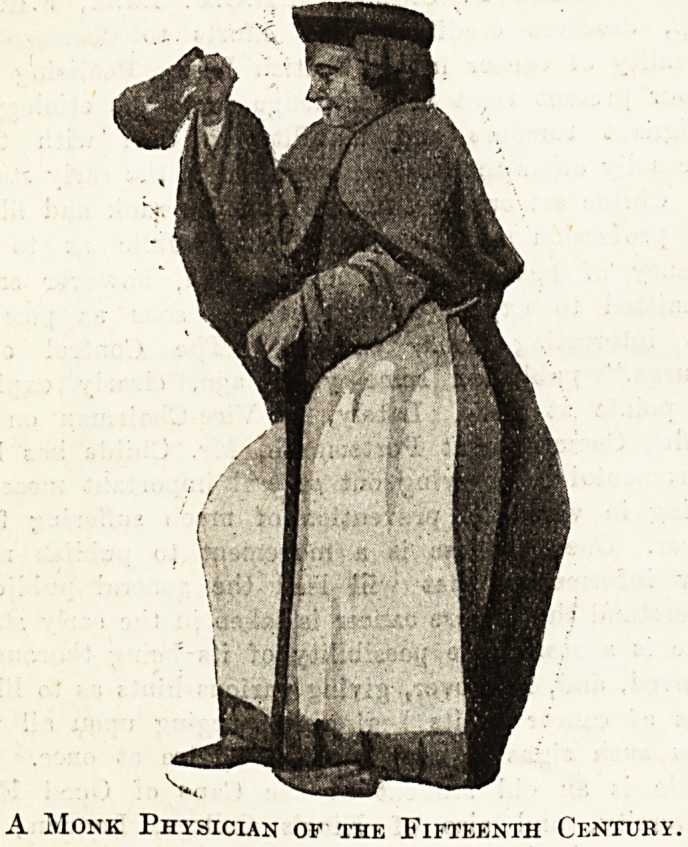


**Figure f2:**
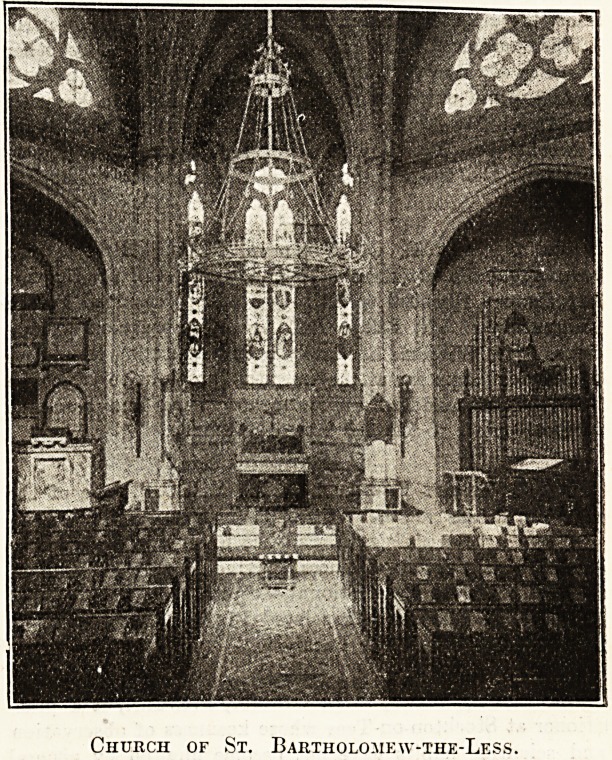


**Figure f3:**
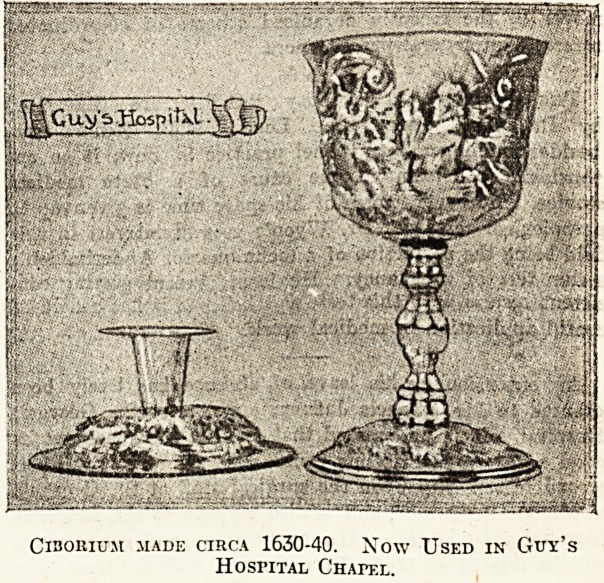


**Figure f4:**
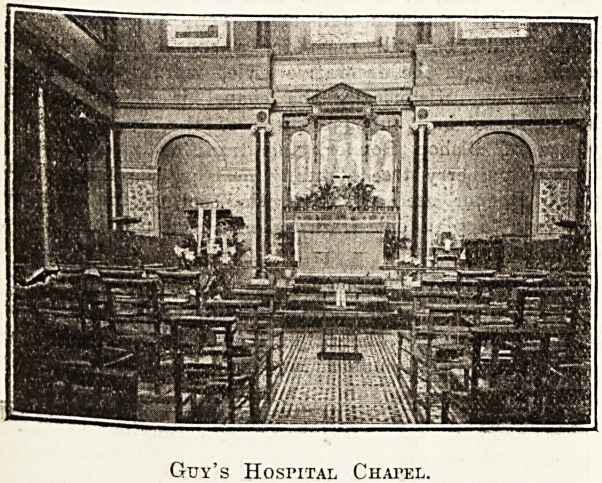


**Figure f5:**
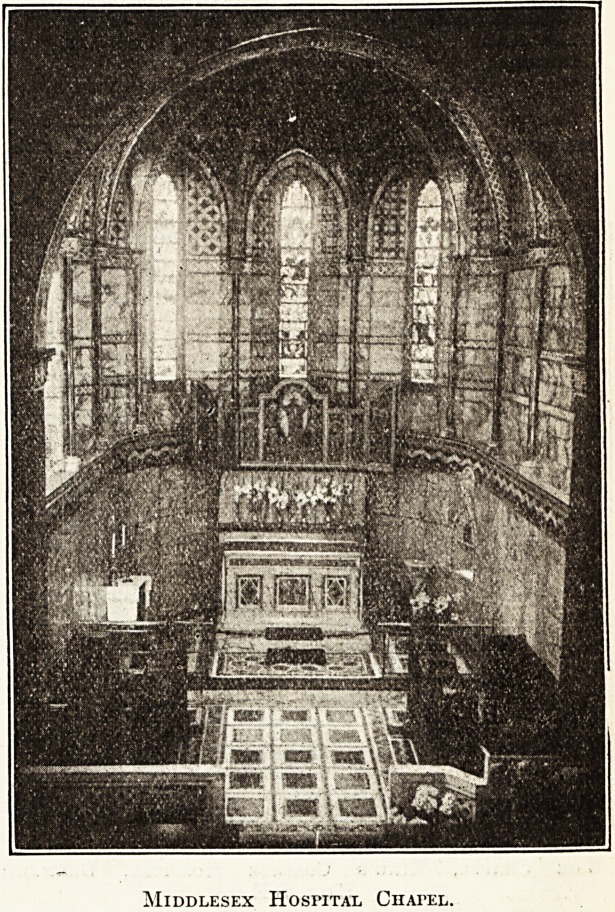


**Figure f6:**
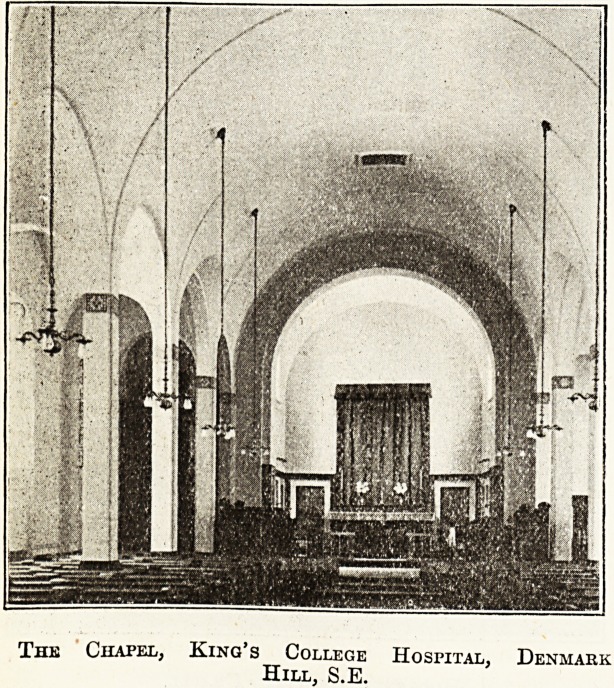


**Figure f7:**